# HMGB1 mediates lipopolysaccharide-induced macrophage autophagy and pyroptosis

**DOI:** 10.1186/s12860-023-00464-7

**Published:** 2023-01-19

**Authors:** Jiawei Shang, Feng Zhao, Yongmei Cao, Feng Ping, Wei Wang, Yingchuan Li

**Affiliations:** 1 Department of Critical Care Medicine, Shanghai Jiaotong University Affiliated Sixth People’s Hospital, Shanghai, 200233 People’s Republic of China; 2 Department of Critical Care Medicine, Fudan University Huashan Hospital, Shanghai, 200040 China; 3 Department of Critical Care Medicine, School of Medicine, Shanghai Tenth People’s Hospital, Tongji University, Shanghai, 200072 China

**Keywords:** Lipopolysaccharide, Macrophages, Autophagy, Pyroptosis, HMGB1, Subcellular localization

## Abstract

Autophagy and pyroptosis of macrophages play important protective or detrimental roles in sepsis. However, the underlying mechanisms remain unclear. High mobility group box protein 1 (HMGB1) is associated with both pyroptosis and autophagy. lipopolysaccharide (LPS) is an important pathogenic factor involved in sepsis. Lentivirus-mediated HMGB1 shRNA was used to inhibit the expression of HMGB1. Macrophages were treated with acetylation inhibitor (AA) to suppress the translocation of HMGB1 from the nucleus to the cytosol. Autophagy and pyroptosis-related protein expressions were detected by Western blot. The levels of caspase-1 activity were detected and the rate of pyroptotic cells was detected by flow cytometry. LPS induced autophagy and pyroptosis of macrophages at different stages, and HMGB1 downregulation decreased LPS-induced autophagy and pyroptosis. Treatment with acetylation inhibitor (anacardic acid) significantly suppressed LPS-induced autophagy, an effect that was not reversed by exogenous HMGB1, suggesting that cytoplasmic HMGB1 mediates LPS-induced autophagy of macrophages. Anacardic acid or an anti-HMGB1 antibody inhibited LPS-induced pyroptosis of macrophages. HMGB1 alone induced pyroptosis of macrophages and this effect was inhibited by anti-HMGB1 antibody, suggesting that extracellular HMGB1 induces macrophage pyroptosis and mediates LPS-induced pyroptosis. In summary, HMGB1 plays different roles in mediating LPS-induced autophagy and triggering pyroptosis according to subcellular localization.

**Supplementary Information:**

The online version contains supplementary material available at 10.1186/s12860-023-00464-7.

## Introduction

The pathophysiology of sepsis involves dysregulation of the inflammatory response, and the imbalance between pro- and anti-inflammatory mediators contributes to the deterioration of sepsis [1, 2]. Monocytes/macrophages are non-specific immune cells that play important roles in monitoring and defense. In the early stage of sepsis, monocytes/macrophages are activated and release a large number of inflammatory cytokines, resulting in an uncontrolled inflammatory response and dysregulation of immune functions [3].

Pyroptosis is a programmed cell death process characterized by the release of inflammatory cytokines. It can be overactivated in sepsis and result in septic shock, multiple organ dysfunction syndrome, or increased risk of secondary infection [4, 5]. A recent study indicated that liver macrophages undergo pyroptosis in an inflammasome-dependent manner during sepsis, contributing to organ dysfunction [6]. Macrophages can also undergo autophagy, another programmed cell death process, during sepsis. Autophagy can play a protective role in sepsis by negatively regulating the abnormal activation of macrophages and suppressing the activation of inflammasomes and the release of inflammatory factors [7]. However, excessive autophagy can aggravate the inflammatory response by leading to autophagic death of macrophages [7].

The specific pathway mediating the processing of macrophages is important in sepsis, although the underlying mechanisms remain unclear. High mobility group box protein 1 (HMGB1), a critical proinflammatory mediator, is involved in both pyroptosis and autophagy [8]. HMGB1 interacts with receptor for advanced glycation end products (RAGE) to initiate HMGB1 endocytosis, which in turn triggers the release of cathepsin B from ruptured lysosomes, followed by pyroptosome formation and caspase-1 activation during macrophage pyroptosis [9]. HMGB1 induces autophagy through multiple pathways in cancer, and loss of HMGB1 in macrophages results in the suppression of autophagy [10, 11]. However, the mechanisms underlying the seemingly contradictory roles of HMGB1 are unknown.

In this study, we investigated the role of HMGB1 in lipopolysaccharide (LPS)-induced autophagy and pyroptosis of macrophages. The results indicate that HMGB1 plays different roles in mediating LPS-induced autophagy and triggering pyroptosis, according to subcellular localization.

## Materials and Methods

### Cell lines and cell culture

Mouse mononuclear RAW264.7 macrophages were purchased from ATCC (Manassas, VA, USA) and cultured in Dulbecco's Modified Eagle's medium supplemented with 10% fetal bovine serum (Thermo Fisher Scientific, Waltham, MA, USA) at 37 °C and 10% CO_2_. LPS (Sigma-Aldrich, St. Louis, MO, USA) or recombinant mouse HMGB1 protein (Novus Biologicals, Centennial, CO, USA) was added into the medium to stimulate RAW264.7 cells at the indicated concentration. 5 mM ATP was added for 1 h before subsequent experiments.

### Western blot analysis

Soluble protein in the culture supernatant was precipitated with 7.2% trichloroacetic acid plus 0.15% sodium deoxycholate. Cells were lysed on ice in RIPA lysis buffer (Thermo Fisher Scientific) supplemented with a protease inhibitor cocktail (Thermo Fisher Scientific). Equal amounts of protein (30 μg) were then separated by 10% –15% sodium dodecyl sulfate polyacrylamide gel electrophoresis and transferred onto polyvinylidene difluoride membranes. The membranes were blocked with 5% fat-free dry milk in Tris Buffered Saline with Tween 20 for 1 h and incubated with primary antibodies, including anti-IL-1β (Cell Signaling Technology, Danvers, MA, USA) and anti-caspase-1 (Santa Cruz Biotech, Dallas, TX, USA) for soluble proteins, and anti-IL-1β, anti-caspase-1, anti-LC3, anti-HMGB1, anti-GAPDH (all from Santa Cruz Biotech) and anti-Gasdermin D (GSDMD) (Abcam, Cambridge, UK) for cytoplasmic proteins for 14 h at 4 °C. The blots were washed and incubated with HRP-conjugated secondary antibodies (Santa Cruz Biotech) for 1 h at room temperature. The blots were cut prior to hybridisation with antibodies during blotting. Protein signals were visualized using an ECL kit (Thermo Fisher Scientific), followed by imaging using the Bio-Rad imaging system (Bio-Rad, Hercules, CA, USA).

### Caspase-1 activity analyses

Caspase-1 activity was determined using the Caspase 1 Activity Assay Kit (Beyotime, China) following the manufacturer’s instructions. Briefly, 50 μg of total cytosolic protein was incubated with 20 nmol Ac-YVAD-pNA overnight at 37 °C. Caspase-1 activity was evaluated by the production of pNA, which was determined by measuring absorbance at 405 nm using a spectrophotometer (Thermo Fisher Scientific).

### Flow cytometry analysis of cell pyroptosis

Cells were incubated with FAM-labeled caspase-1 FLICA (Bio-Rad, Hercules, CA, USA) at 37 °C for 1 h. Cells were fixed with 4% paraformaldehyde and then stained with TMR red-labeled In-Situ Cell Death Detection reagent (Roche Applied Science, Indianapolis, IN, USA) for 1 h. The cells were analyzed by flow cytometry using a FACScalibur flow cytometer (BD Biosciences, San Jose, CA, USA). Background and auto-fluorescence were determined using isotype controls. The double-stained cells were counted as pyroptotic cells, and the rate of pyroptosis was calculated.

### Cell transfection

RAW264.7 cells were cultured in 12-well plates for 24 h before transfection. Cells were transfected with HMGB1 shRNA lentiviral particles (Santa Cruz Biotechnology) using Polybrene. After 48 h of transfection, the efficiency of HMGB1 knockdown was confirmed by real-time PCR and western blotting.

### Real-time PCR

Total RNA was isolated using the Trizol reagent (Ambion, USA). cDNA was prepared using a Verso cDNA synthesis kit (Thermo Fisher Scientific) following the manufacturer’s protocol with a total reaction volume of 20 μl. Real-time PCR was performed using a SYBR green master mix qPCR kit (Thermo Fisher Scientific) on the ABI 7500 real-time PCR system. The primers for HMGB1 were 5′-GCTGACAAGGCTCGTTATGAA-3′ (forward) and 5′ -CCTTTGATTTTGGGGCGGTA-3′ (reverse), and those for GAPDH were 5′ -AGGTCGGTGTGAACGGATTTG-3′(forward) and 5′ -GGGGTCGTTGATGGCAACA-3′ (reverse). Relative mRNA expression was normalized to GAPDH using the 2^−ΔΔCt^ method.

### Immunofluorescence confocal microscopy

RAW264.7 cells were cultured on glass coverslips in 12-well plates and treated with anacardic acid (AA, Sigma-Aldrich) (25 mmol/l) or anti-HMGB1 antibody (10 μg/ml) for 12 h. Then, the coverslips were fixed with 2% paraformaldehyde for 15 min at room temperature and permeabilized with 0.1% Triton X-100 for 10 min at room temperature. After incubation with anti-HMGB1 antibody (1:500) overnight at 4 °C, cells were incubated with Alexa Fluor Plus 555 conjugated secondary antibody (Thermo Fisher Scientific) (1:200) for 1 h. 4',6-diamidino-2-phenylindole was used for nuclear staining. Images were acquired with a confocal microscope (Zeiss LSM 510 Meta; Carl Zeiss).

### Statistical analysis

The data from at least three experiments are presented as the mean ± SD. The significance of differences between multiple groups and between two groups was determined using one-way ANOVA followed by Tukey’s post-hoc test and the two-tailed Student's t-test, respectively. *P* < 0.05 was considered statistically significant.

## Results

### LPS induces autophagy and pyroptosis of macrophages at different stages

**Fig. 1:**
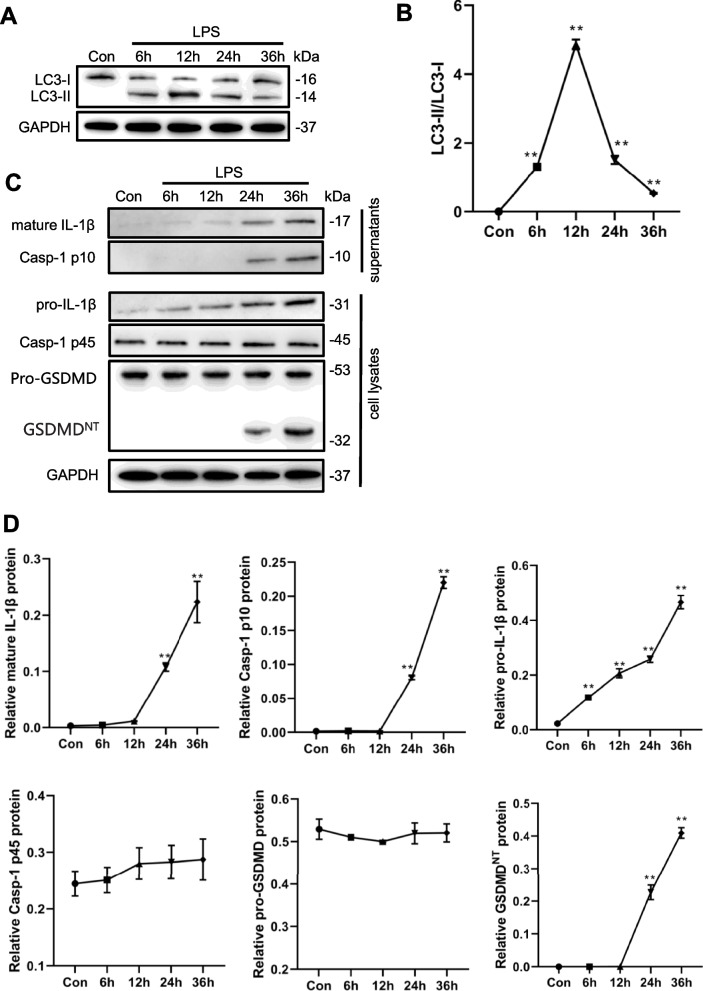
LPS increases autophagy and pyroptosis-related proteins levels. Mouse mononuclear macrophage RAW264.7 were treated with LPS (1 μg/ml) for 0-36 h. **A**, **B**. Western blot analysis of autophagy-related protein (LC3) expression. **C**, **D**. Western blot analysis of the pyroptosis-related protein expression in culture supernatants and cell lysates. **P < 0.01 versus the control group

**Fig. 2:**
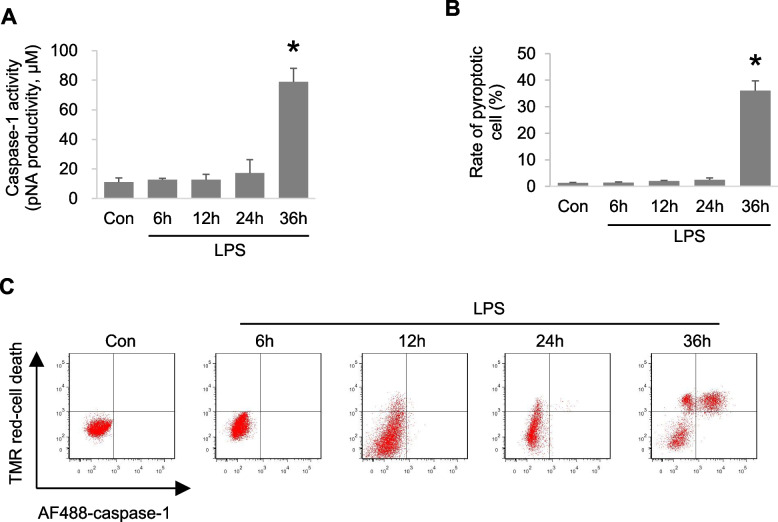
LPS induces macrophage autophagy and pyroptosis. Mouse mononuclear macrophage RAW264.7 were treated with LPS (1 μg/ml) for 0-36 h. **A**. The levels of caspase-1 activity were detected. **B**, **C**. The rate of pyroptotic cell was detected by flow cytometry. *P < 0.05 versus the control group

To evaluate the effects of LPS on macrophage autophagy and pyroptosis, mouse mononuclear RAW264.7 macrophages were treated with LPS for different times and analyzed by western blotting. The results showed that the levels of endogenous LC3-II increased significantly at 12 h after LPS treatment and began to decrease after 24 h (Fig. 1A, B). Next, we detected caspase-1 activity and the levels of cleaved caspase-1 and IL-1β. The results showed that mature IL-1β and caspase-1 p10 were released into the culture supernatant after LPS treatment for 24 h and continued to increase at 36 h (Fig. 1C, D). As a pyroptosis inducing protein, GSDMD was also cleaved to release GSDMD -N domain (GSDMD^NT^) after LPS treatment for 24 h and 36 h (Fig. 1C, D). LPS significantly increased caspase-1 activity after 36 h (Fig. 2A). The rate of pyroptotic cells detected by flow cytometry also increased significantly after 36 h of LPS treatment (Fig. 2B, C). These results indicate that LPS induced autophagy and pyroptosis of macrophages at different stages.

#### HMGB1 downregulation blocks LPS-induced autophagy and pyroptosis of macrophages

**Fig. 3:**
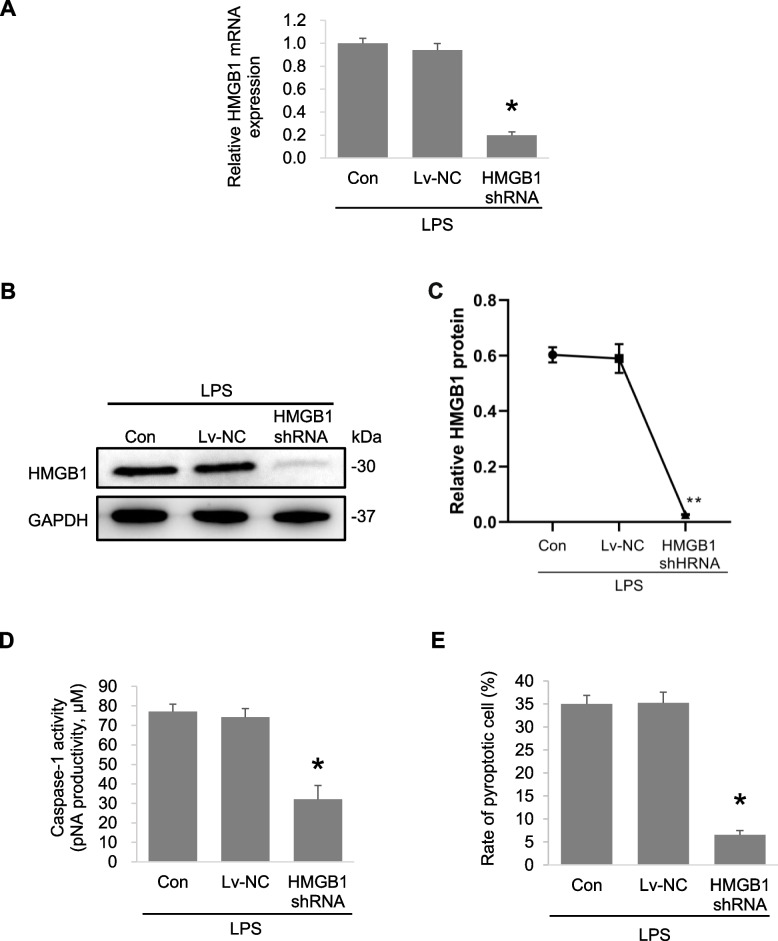
Knockdown of HMGB1 mitigates the LPS-induced macrophage autophagy and pyroptosis. Macrophage RAW264.7 were transfected with Lentivirus-mediated HMGB1 shRNA or non-specific shRNA (Lv-NC) and then treated with LPS (1 μg/ml) for 12 h. HMGB1 expression levels were detected by Realtime PCR (**A**) and western blot (**B**, **C**). **D**. The levels of caspase-1 activity were detected after treatment with LPS for 36 h. E. The rate of pyroptotic cell was detected by flow cytometry after treatment with LPS for 36 h. *P < 0.05, *P* < 0.01 versus the control + LPS group

**Fig. 4:**
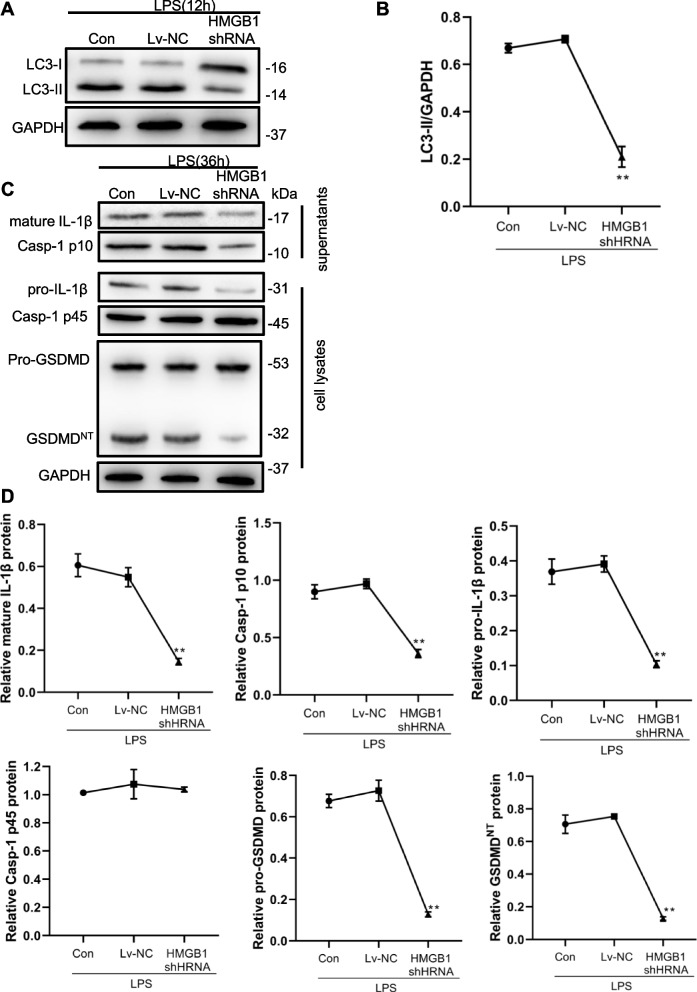
Knockdown of HMGB1 decreases autophagy and pyroptosis-related proteins levels. Macrophage RAW264.7 were transfected with Lentivirus-mediated HMGB1 shRNA or non-specific shRNA (Lv-NC) and then treated with LPS (1 μg/ml) for 12 h. Autophagy-related protein (LC3) expression (**A**, **B**) and pyroptosis-related protein expression (**C**, **D**) were detected by western blot after treatment with LPS for 12 h or 36 h respectively. *P < 0.05, *P* < 0.01 versus the control + LPS group

To explore the role of HMGB1 in LPS-induced autophagy and pyroptosis of macrophages, lentivirus-mediated HMGB1 shRNA was used to inhibit the expression of HMGB1 in mouse monocyte RAW264.7 macrophages. Real-time PCR and western blotting showed that HMGB1 expression was significantly downregulated by shRNA in macrophages treated with LPS (Fig. 3A-C). HMGB1 shRNA also inhibited caspase-1 activity and pyroptosis of macrophages (Fig. 3D, E). Besides, HMGB1 downregulation significantly suppressed the LPS-induced increase of endogenous LC3-II levels (12 h) (Fig. 4A, B). Therefore, we can draw that LPS induced an increase of LC3-II level and HMGB1 knockdown decreases LC3-II levels in cells treated with LPS according to the experimental results from Figs. 1A, B and Fig. 4A, B. The relative levels of mature IL-1β, caspase-1 p10 and cleavage of the GSDMD-N domain, caspase-1 activity, and the rate of pyroptosis were significantly decreased in macrophages transfected with HMGB1 shRNA compared with the controls after LPS treatment for 36 h (Fig. 4C, D). These results indicate that HMGB1 downregulation blocked LPS-induced autophagy and pyroptosis of macrophages.

#### Cytoplasmic HMGB1 mediates LPS-induced autophagy of macrophages

**Fig. 5:**
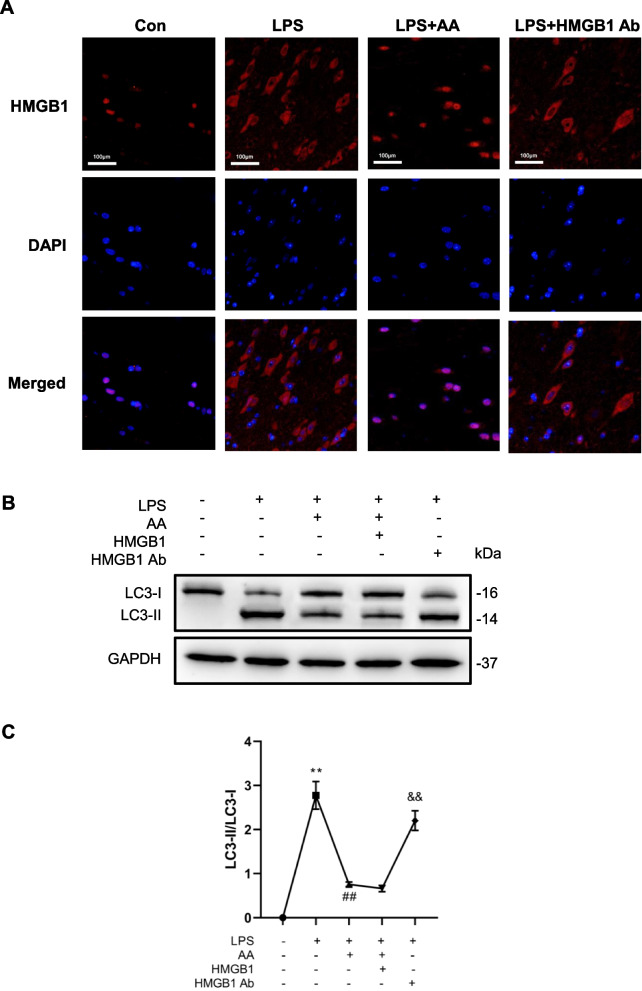
Cytoplasm HMGB1 mediates LPS-induced autophagy of macrophages. **A**. Macrophage RAW264.7 were pretreated with 25 mmol/l anacardic acid (AA) and then treated with LPS (1 μg/ml) or LPS combined anti-HMGB1 antibodies (10 μg/ml) for 12 h. HMGB1 expression and location were detected by immunofluorescence. **B**, **C**. Macrophage RAW264.7 were pretreated with AA and then treated with LPS or LPS combined HMGB1 (500 ng/ml) or anti-HMGB1 antibodies for 12 h. LC3 expression were detected by western blot. **P < 0.01 versus the control group. ^##^*P* < 0.01 versus the LPS group. ^&&^*P* < 0.01 versus the LPS + AA + HMGB1 group

HMGB1 is present in the nucleus and cytoplasm of cells, and is released from cells during infection and sterile tissue injury [12]. It may function depending on its localization [13]. To determine whether HMGB1 with different subcellular localizations regulates LPS-induced autophagy of macrophages, we treated macrophages with acetylation inhibitor (AA) to suppress the translocation of HMGB1 from the nucleus to the cytosol. As shown in Fig. 5A, LPS treatment promoted the translocation of HMGB1 from the nucleus to the cytosol, and AA inhibited this translocation, whereas anti-HMGB1 antibody had no significant effect. LPS induced an increase in LC3II protein level and AA significantly suppressed the LPS-induced increase of endogenous LC3-II levels, an effect that was not reversed by exogenous HMGB1 (Fig. 5B-C). An anti-HMGB1 antibody was used to block extracellular HMGB1, which had no effect on LC3-II levels (Fig. 5B-C). These results demonstrate that HMGB1 regulates LPS-induced macrophage autophagy in the cytoplasm but not in the nucleus or the extracellular compartment.

#### Extracellular HMGB1 induces macrophage pyroptosis

**Fig. 6:**
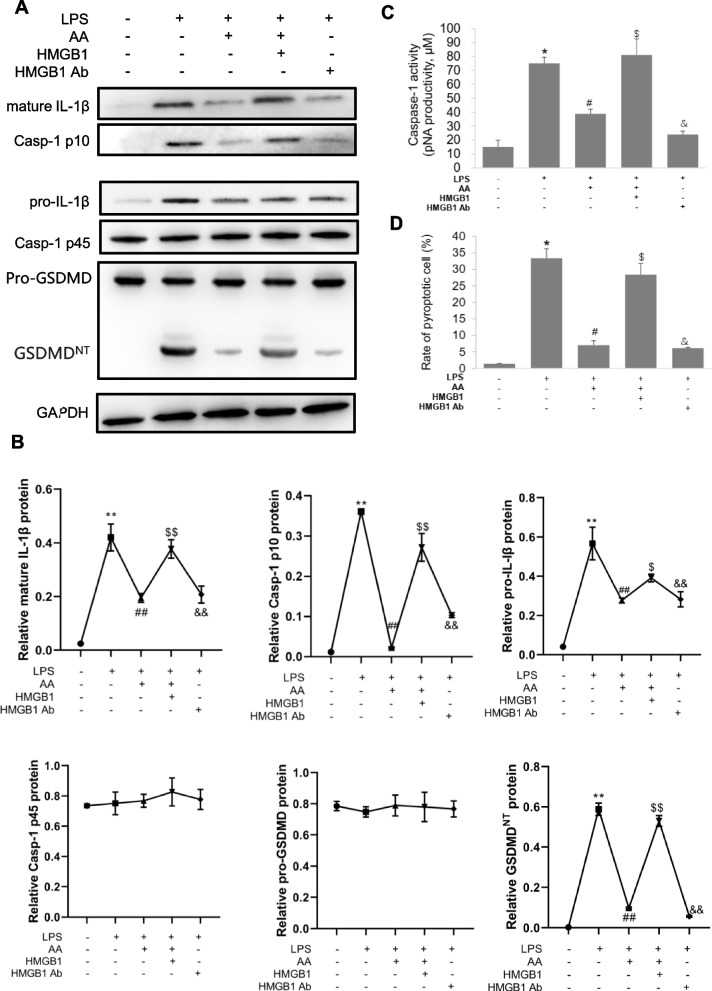
Extracellular HMGB1 up-regulates pyroptosis-related proteins levels in macrophage. Macrophage RAW264.7 were pretreated with AA and then treated with LPS or LPS combined HMGB1 or anti-HMGB1 antibodies for 36 h. **A**, **B**. The pyroptosis-related protein expressions were detected by western blot. **C**. The levels of caspase-1 activity were detected. **D**. The rate of pyroptotic cell was detected by flow cytometry. *P < 0.05, **P < 0.01 versus the control group. ^#^*P* < 0.05, ^##^*P* < 0.01 versus the LPS group. ^$^*P* < 0.05, ^$$^*P* < 0.01 versus the LPS + AA group. ^&^*P* < 0.05, ^&&^*P* < 0.01 versus the LPS + AA + HMGB1 group

**Fig. 7:**
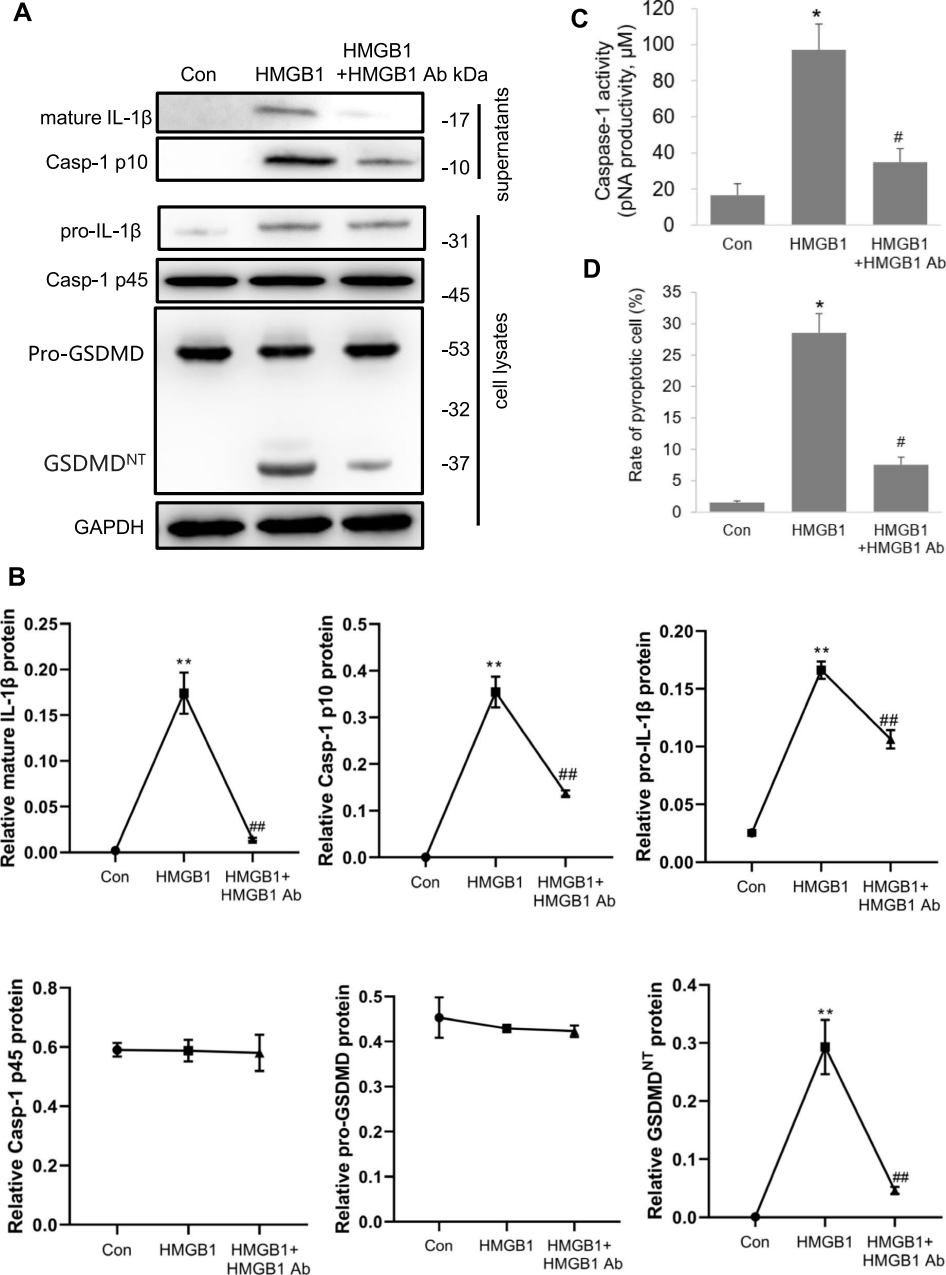
Extracellular HMGB1 induces macrophage pyrolysis. Macrophage RAW264.7 were treated with HMGB1 (500 ng/ml) or HMGB1 combined anti-HMGB1 antibodies (1 μg/ml). **A**, **B**. The pyroptosis-related protein expressions were detected by western blot. **C**. The levels of caspase-1 activity were detected. **D**. The rate of pyroptotic cell was detected by flow cytometry. *P < 0.05, **P < 0.01 versus the control group. ^#^*P* < 0.05, ^##^*P* < 0.01 versus the HMGB1 group

Next, we investigated the relationship between HMGB1 with different subcellular localizations and LPS-induced pyroptosis. As shown in Fig. 6A–D, treatment with AA or anti-HMGB1 antibody inhibited LPS-induced pyroptosis of macrophages. Treatment with HMGB1 alone induced pyroptosis of macrophages and was inhibited by anti-HMGB1 antibody (Fig. 7A, B). The results indicate that extracellular HMGB1 can induce macrophage pyroptosis alone and also mediate LPS-induced pyroptosis.

## Discussion

**Fig. 8:**
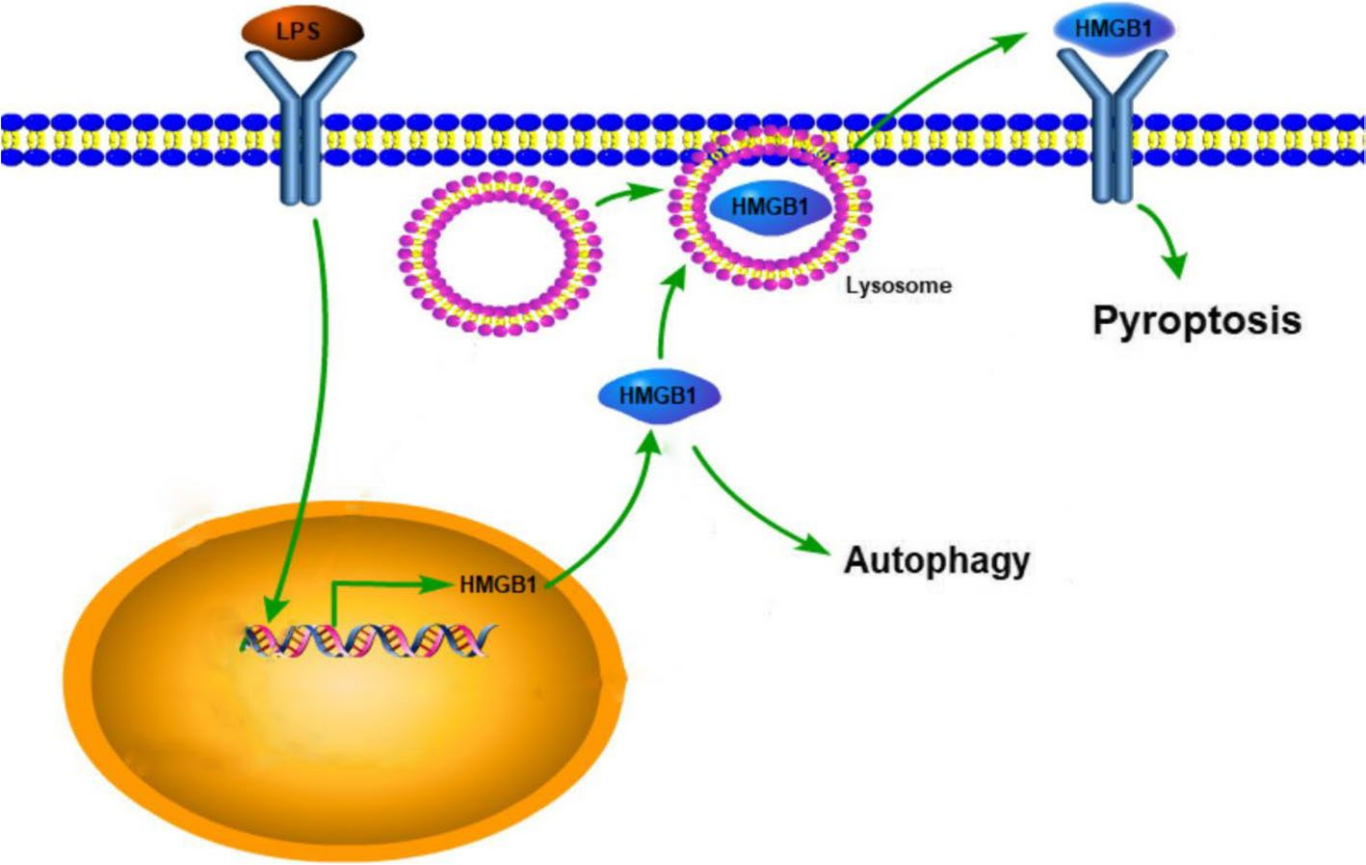
The flow of the LPS-HMGB1 axis. LPS-induced autophagy and pyroptosis of macrophages occur at different stages. HMGB1 played an important role in both LPS-induced programmed cell death processes in a manner dependent on its subcellular localization

LPS, an important pathogenic factor involved in sepsis, induces macrophage pyroptosis and autophagy [7, 14]. In the current study, we demonstrated that LPS-induced autophagy and pyroptosis of macrophages occur at different stages. HMGB1 played an important role in both LPS-induced programmed cell death processes in a manner dependent on its subcellular localization (Fig. 8).

LPS can induce macrophage autophagy through toll-like receptor 4 (TLR4)-dependent pathways [15]. Evidence suggests that autophagy plays a protective role in sepsis by directly mediating pathogen clearance [16] and by modulating the release of cytokines [17]. Autophagy inhibition is closely related to organ dysfunction in sepsis [18, 19]. However, LPS-induced autophagy does not abolish late immune dysfunction and tissue damage. Pyroptosis, another form of programmed cell death in macrophages, may help explain this phenomenon. LPS triggers caspase-11-dependent pyroptosis of macrophages, which in turn enhances inflammation [20].

The present results showed that LPS induced both autophagy and pyroptosis of macrophages, although autophagy occurred earlier than pyroptosis. This indicates that macrophage autophagy may protect cells from the effects of short-term LPS stimulation, whereas, continuous exposure to LPS may cause damage by inducing pyroptosis of macrophages.

HMGB1, a downstream inflammatory mediator of LPS, is considered to be involved in both autophagy and pyroptosis. Previous studies suggesting that HMGB1 affect autophagy by interaction with Beclin 1 [21, 22]. We performed some preliminary experiments and found that HMGB1 was not colocalized with autophagosomes. In this study, our results indicated that enhanced autophagy may promote the secretion of HMGB1 and promote pyroptosis. HMGB1 downregulation blocked LPS-induced autophagy and pyroptosis of macrophages, suggesting that the effects of LPS on autophagy and pyroptosis were mediated by HMGB1.

LPS promotes the migration of HMGB1 from the nucleus to the cytoplasm and its secretion to the extracellular compartment [23]. Studies in tumor cells show that HMGB1 can induce autophagy in different compartments, including the nucleus, cytoplasm, and extracellular fluid [10]. Acetylation of lysine residues in HMGB1 is necessary for its translocation from the nucleus to the cytoplasm [24]. We used AA treatment to inhibit HMGB1 translocation, and found that LPS-induced autophagy was decreased, and exogenous HMGB1 did not increase autophagy. This suggests that, unlike its effect in tumors, cytoplasmic HMGB1 plays a major role in regulating LPS-induced autophagy in macrophages. HMGB1 migration from the nucleus to the cytoplasm was observed after 4 h of LPS simulation [12], which was consistent with the results showing that macrophage autophagy increased at 6 h after LPS treatment (Fig. 1A).

HMGB1 induces macrophage pyroptosis through two pathways. On the one hand, HMGB1 directly interacts with RAGE in macrophages to trigger endocytosis, thereby initiating a cascade of cellular events, including the release of cathepsin B from ruptured lysosomes, the formation of inflammasomes, and the activation of caspase-1 [9]. Furthermore, HMGB1 binds to LPS and delivers extracellular LPS to the cytosol of macrophages, where LPS activates caspase-11 and downstream caspase-1 [20]. The present results showed that either AA or anti-HMGB1 antibody inhibited LPS-induced pyroptosis of macrophages, suggesting that extracellular HMGB1 was involved in LPS-induced pyroptosis. Moreover, treatment with HMGB1 alone induced pyroptosis of macrophages and was inhibited by an anti-HMGB1 antibody, suggesting that HMGB1 triggers pyroptosis via the first RAGE pathway. Secretion of HMGB1 by LPS-activated monocytes is a late event. HMGB1 secretion is still increasing at 18 and 30 h [25], which explains the finding that LPS induced pyroptosis later than autophagy.

HMGB1 is secreted from the cytoplasm to the outside of the cell by lysosome-mediated exocytosis [25]. Recent studies show that autophagy facilitates the active export of additional unconventionally secreted proteins, including HMGB1, in a process called secretory autophagy [26]. This indicates that enhanced autophagy may promote the secretion of HMGB1 and promote pyroptosis, which may explain the paradoxical effects of autophagy activators in sepsis [27, 28] and deserves further study.

In summary, the present study demonstrated that HMGB1 promotes LPS-induced autophagy and pyroptosis and plays different roles according to its subcellular localization.

## Supplementary Information

**Additional file 1.**

## Data Availability

The datasets used and/or analyzed during the current study are available from the corresponding author on reasonable request.
