# Supplementary figures and images for: HMGB1 mediates lipopolysaccharide-induced macrophage autophagy and pyroptosis
Source: BMC Mol Cell Biol. 2023 Jan 19;24:2. doi: 10.1186/s12860-023-00464-7 (PMC9854035; doi:10.1186/s12860-023-00464-7)

Figure1A

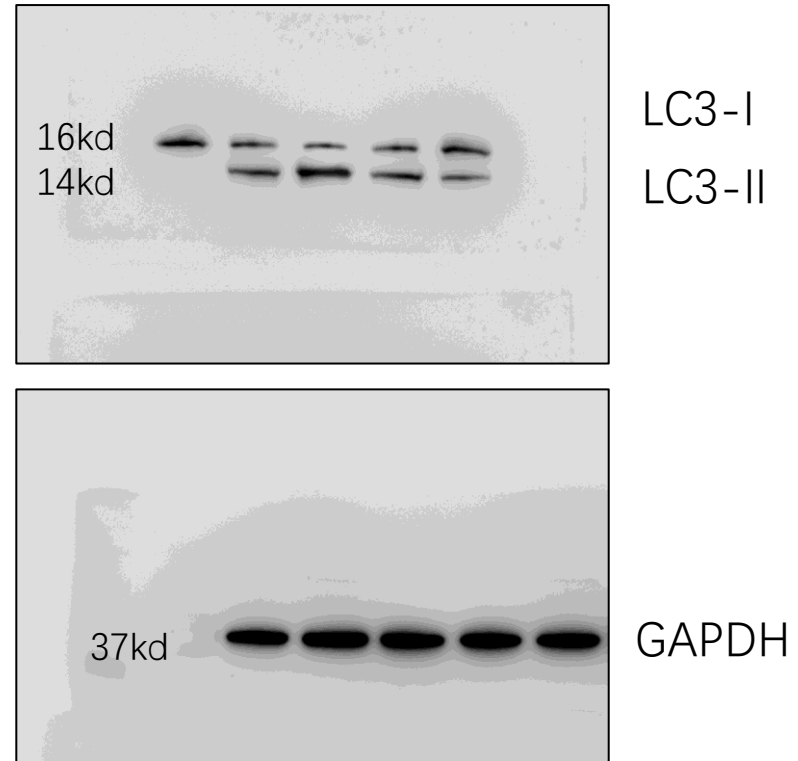

Figure1C

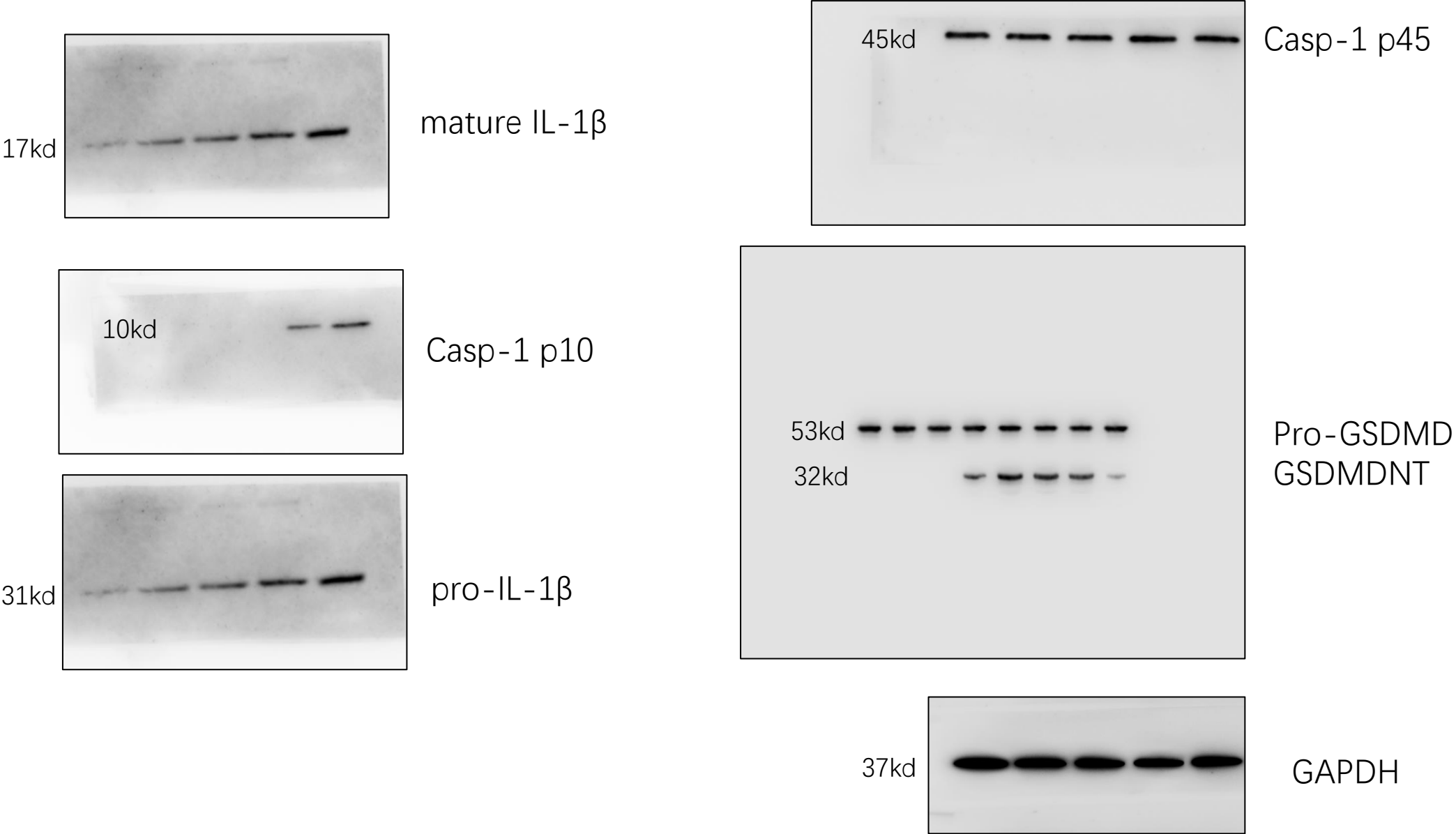

Figure3B

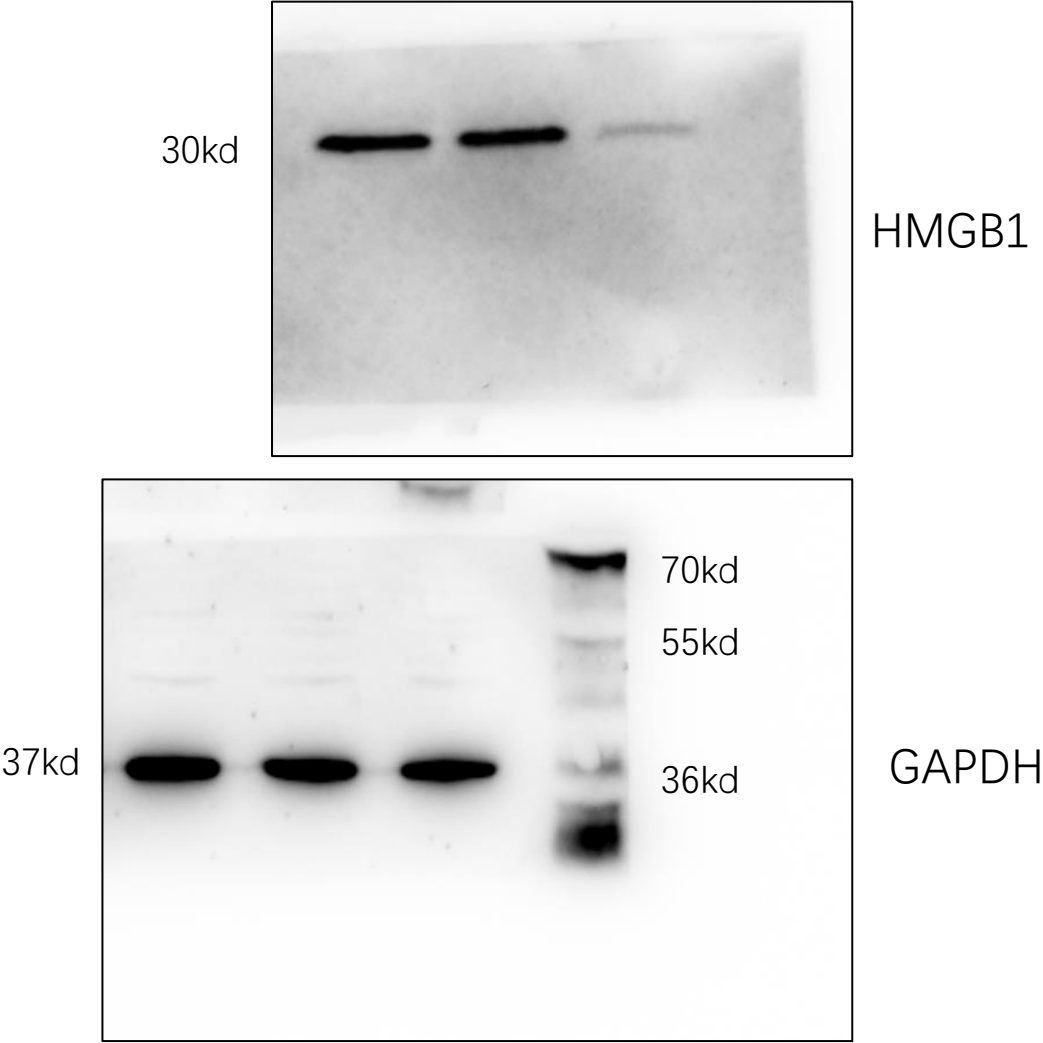

Figure 4A

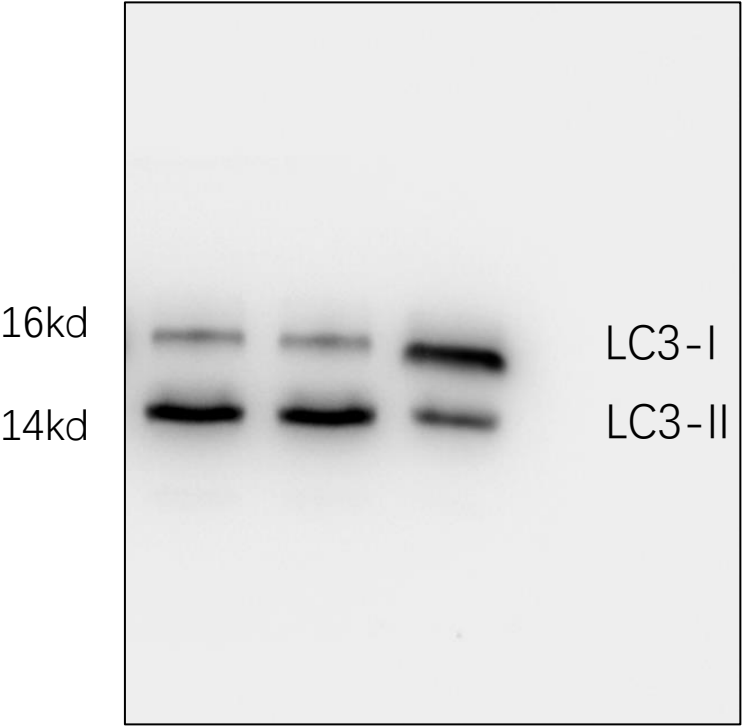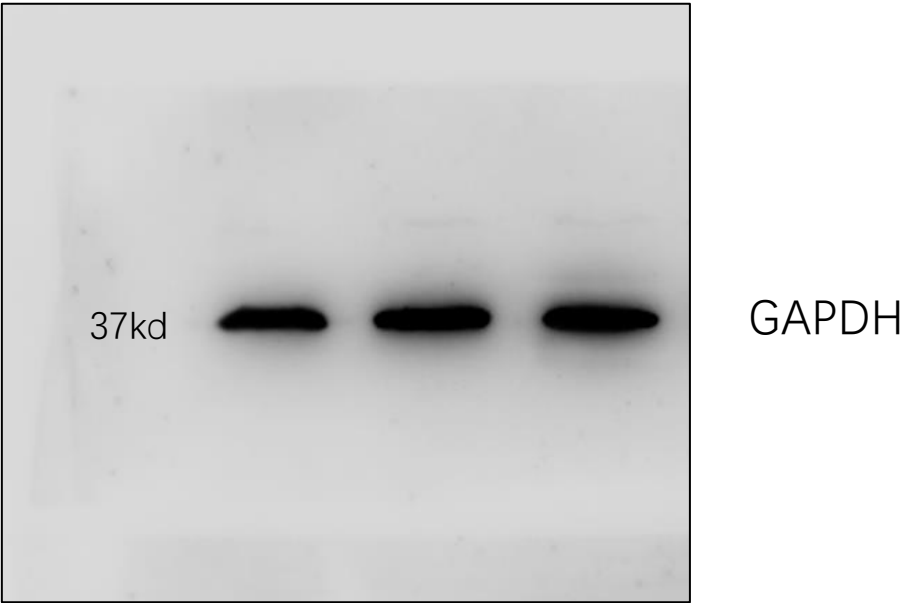

Figure 4C

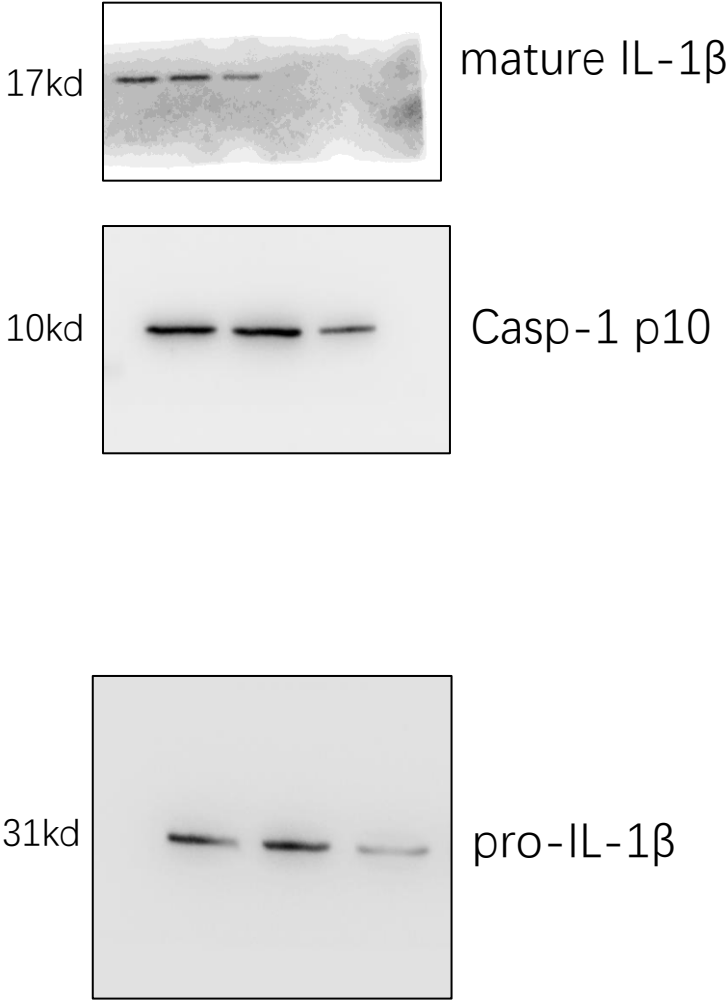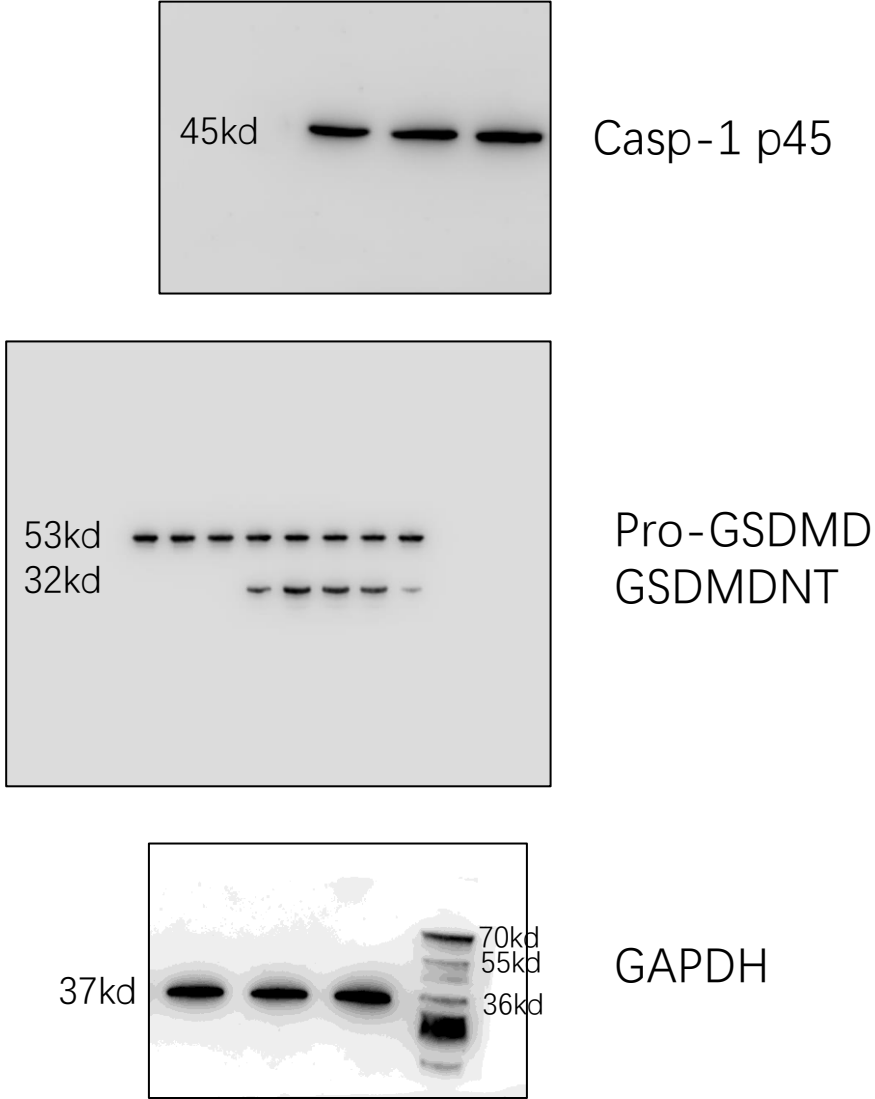

Figure 5B

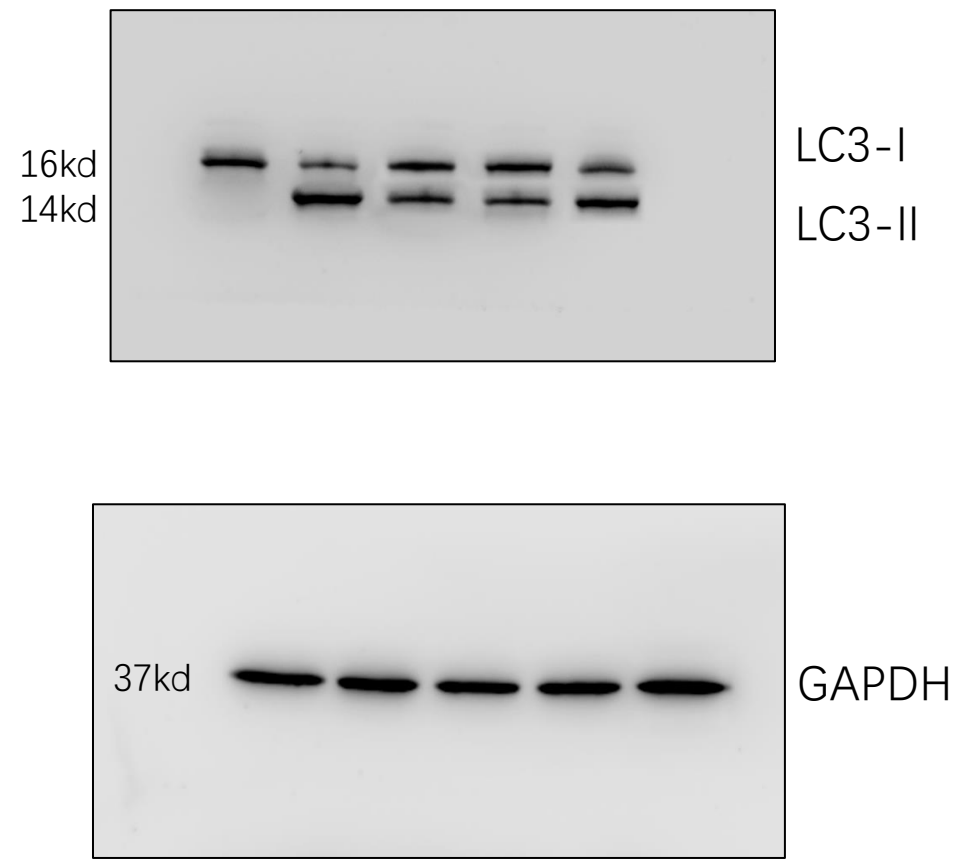

Figure 6A

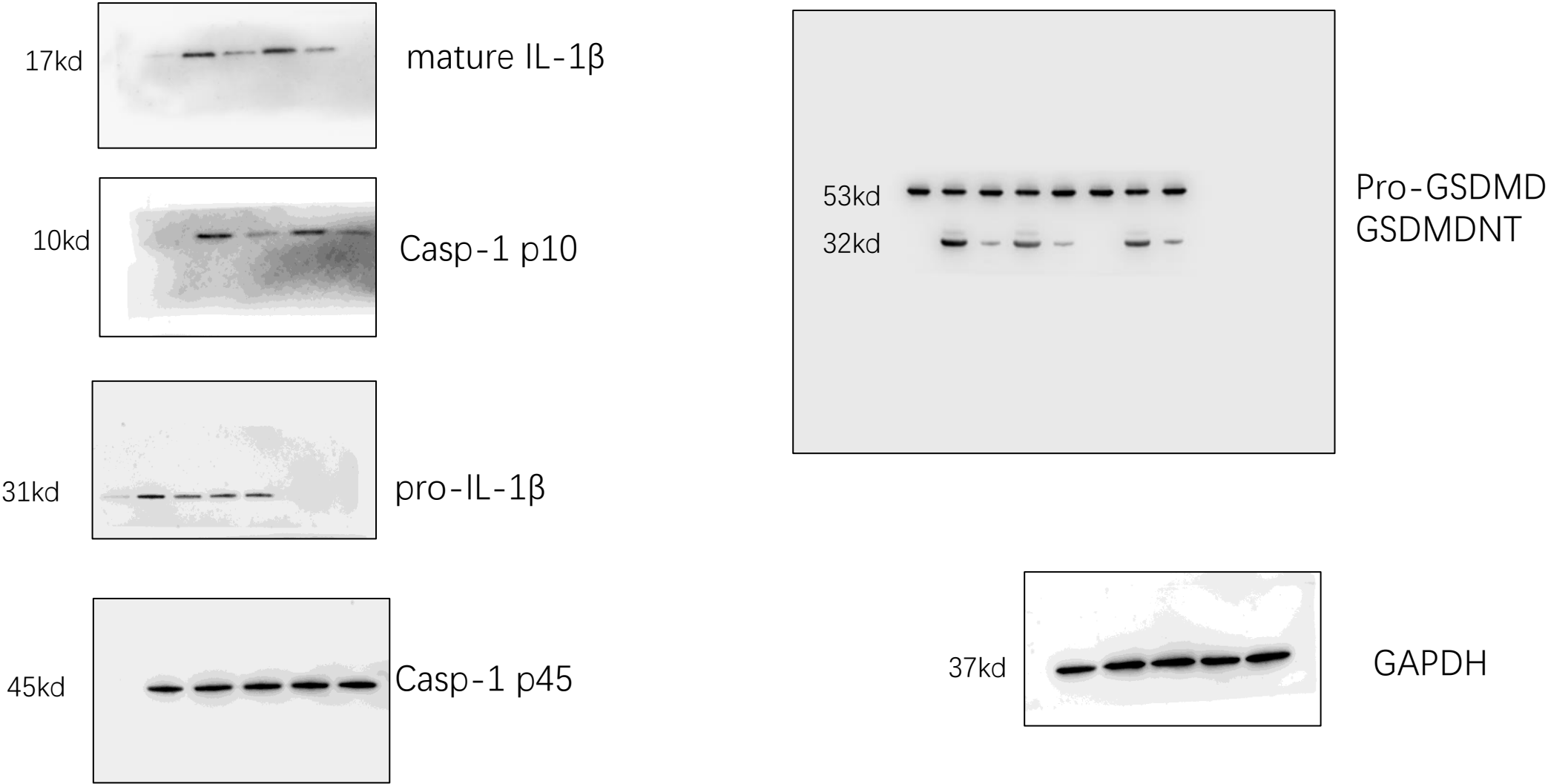

Figure 7A

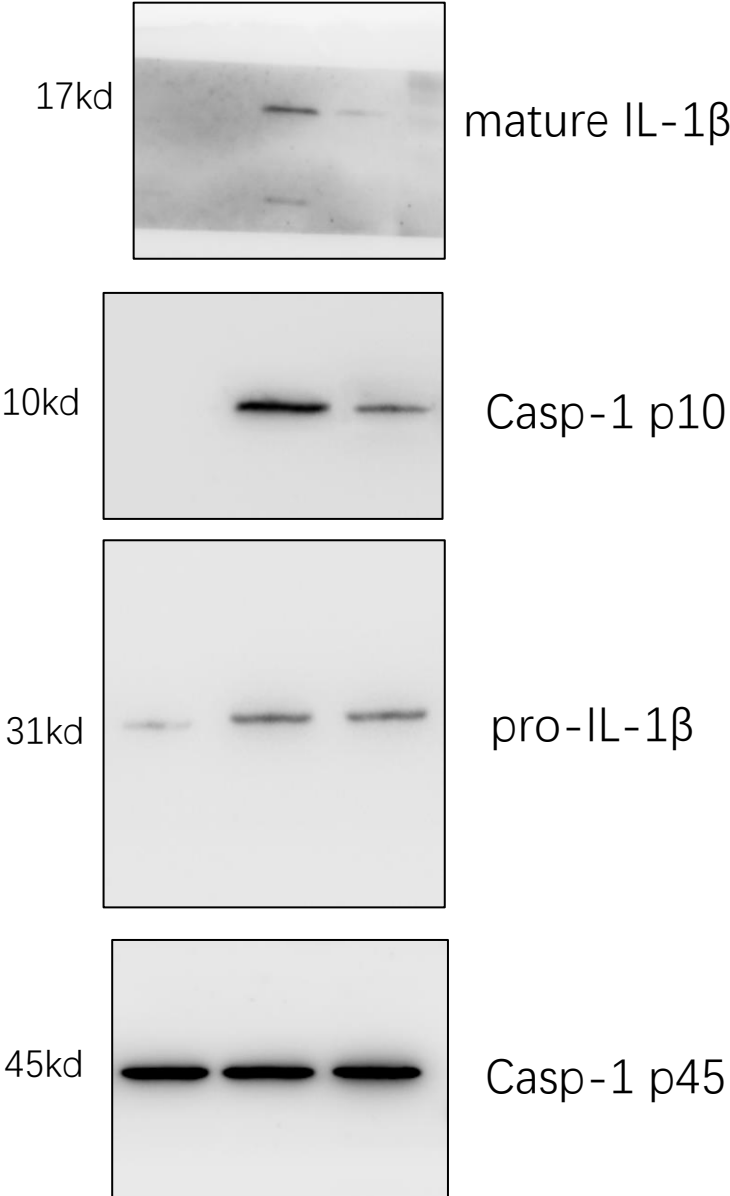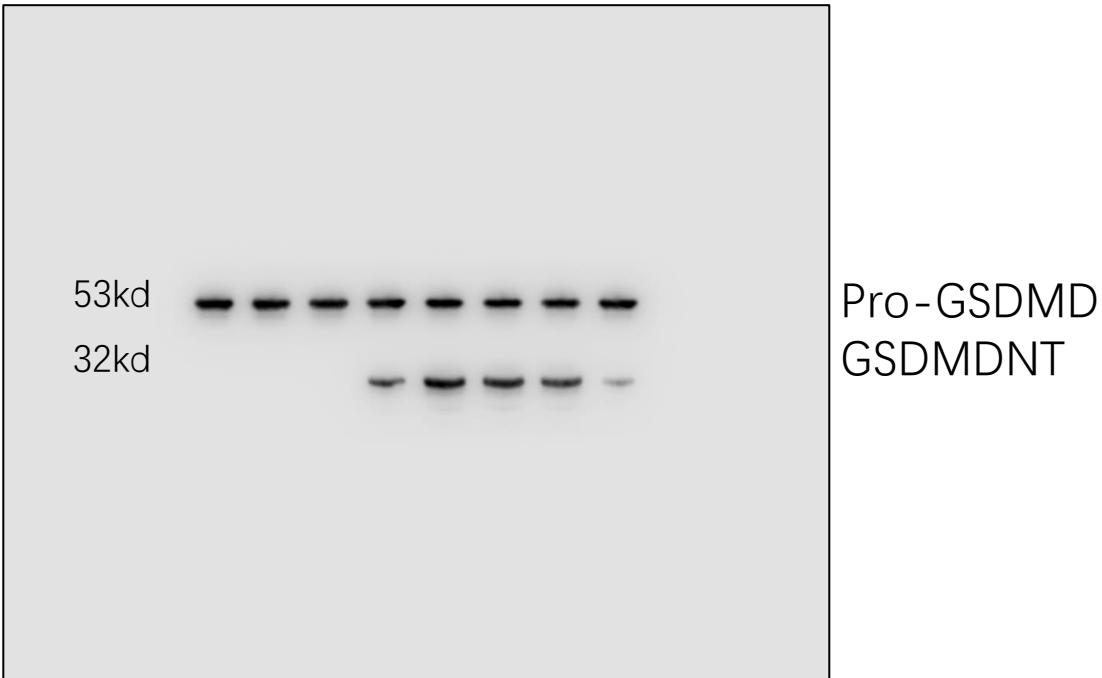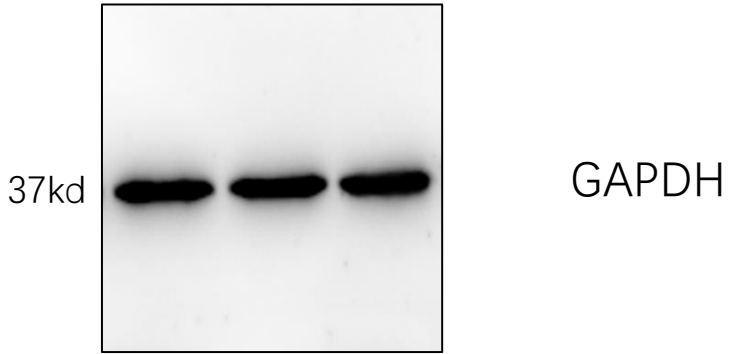

Supplement: Supplementary file 1 — Additional file 1. [file 12860_2023_464_MOESM1_ESM.pdf]
